# Shoulder Surgery Postoperative Immobilization: An International Survey of Shoulder Surgeons

**DOI:** 10.3390/biology12020291

**Published:** 2023-02-11

**Authors:** Michael T. Freehill, Iain R. Murray, Emilio Calvo, Alexandre Lädermann, Uma Srikumaran

**Affiliations:** 1 Department of Orthopaedic Surgery, Stanford University School of Medicine, Redwood City, CA 94305, USA; 2 Edinburgh Orthopaedics, The Royal Infirmary of Edinburgh, Edinburgh EH4 2XU, UK; 3 The University of Edinburgh, Edinburgh EH8 9JU, UK; 4 Shoulder and Elbow Reconstructive Surgery Unit, Orthopaedic Surgery and Trauma Department, Fundación Jiménez Díaz University Hospital, Universidad Autonoma, 28040 Madrid, Spain; 5 La Tour Hospital, University of Geneva, Geneva University Hospitals, 1211 Geneva, Switzerland; 6 Faculty of Medicine, University of Geneva, 1211 Geneva, Switzerland; 7 Division of Orthopaedics and Trauma Surgery, Department of Surgery, Geneva University Hospitals, 1217 Geneva, Switzerland; 8 Department of Orthopaedic Surgery, John Hopkins University School of Medicine, Baltimore, MD 21205, USA

**Keywords:** sling, rehabilitation, Latarjet, Bankart, arthroplasty, prosthesis: reverse, complications, rehabilitation

## Abstract

**Simple Summary:**

No consensus currently exists on immobilization protocols following shoulder surgery. The aim of this study was to identify patterns and types of sling used by surgeons from the United States and Europe for a variety of shoulder surgical procedures and further to identify factors associated with the variations. Four-hundred and ninety-nine surgeons with a median 15 years of experience responded, with 54.7% from the United States and 45.3% from Europe. United States surgeons reported higher abduction pillow sling use than European surgeons, whereas European surgeons reported more simple sling utilization. Increasing experience was negatively correlated with sling duration, meaning more experienced respondents tended to recommend shorter durations of sling use. Considerable variation exists in the immobilization patterns after a variety of shoulder surgical procedures advocated by surgeons with apparent influence from both geographic location and years of clinical experience. Future work is required to establish the most clinically beneficial protocols for immobilization following shoulder surgery.

**Abstract:**

Background: There is currently no consensus on immobilization protocols following shoulder surgery. The aim of this study was to establish patterns and types of sling use for various surgical procedures in the United States (US) and Europe, and to identify factors associated with the variations. Methods: An online survey was sent to all members of the American Shoulder and Elbow Society (ASES) and European Society for Surgery of the Shoulder and Elbow (ESSSE). The survey gathered member data, including practice location and years in practice. It also obtained preferences for the type and duration of sling use after the following surgical procedures: arthroscopic Bankart repair, Latarjet, arthroscopic superior/posterosuperior rotator cuff repair (ARCR) of tears <3 cm and >3 cm, anatomic total shoulder arthroplasty (aTSA) and reverse TSA (rTSA), and isolated biceps tenodesis (BT). Relationships between physician location and sling type for each procedure were analyzed using Fisher’s exact tests and post-hoc tests using Bonferroni-adjusted *p*-values. Relationships looking at years in practice and sling duration preferred were analyzed using Spearman’s correlation tests. Results: In total, 499 surgeons with a median of 15 years of experience (IQR = 9–25) responded, with 54.7% from the US and 45.3% from Europe. US respondents reported higher abduction pillow sling use than European respondents for the following: Bankart repair (62% vs. 15%, *p* < 0.0001), Latarjet (53% vs. 12%, *p* < 0.001), ARCR < 3 cm (80% vs. 42%, *p* < 0.001) and >3 cm (84% vs. 61%, *p* < 0.001), aTSA (50% vs. 21%, *p* < 0.001) and rTSA with subscapularis repair (61% vs. 22%, *p* < 0.001) and without subscapularis repair (57% vs. 17%, *p* < 0.001), and isolated BT (18% vs. 7%, *p* = 0.006). European respondents reported higher simple sling use than US respondents for the following: Bankart repair (74% vs. 31%, *p* < 0.001), Latarjet (78% vs. 44%, *p* < 0.001), ARCR < 3 cm (50% vs. 17%, *p* < 0.001) and >3 cm (34% vs. 13%, *p* < 0.001), and aTSA (69% vs. 41%, *p* < 0.001) and rTSA with subscapularis repair (70% vs. 35%, *p* < 0.001) and without subscapularis repair (73% vs. 39%, *p* < 0.001). Increasing years of experience demonstrated a negative correlation with the duration of sling use after Bankart repair (r = −0.20, *p* < 0.001), Latarjet (r = −0.25, *p* < 0.001), ARCR < 3 cm (r = −0.14, *p* = 0.014) and >3 cm (r = −0.20, *p* < 0.002), and aTSA (r = −0.37, *p* < 0.001), and rTSA with subscapularis repair (r = −0.10, *p* = 0.049) and without subscapularis repair (r = −0.19, *p* = 0.022. Thus, the more experienced surgeons tended to recommend shorter durations of post-operative sling use. US surgeons reported longer post-operative sling durations for Bankart repair (4.8 vs. 4.1 weeks, *p* < 0.001), Latarjet (4.6 vs. 3.6 weeks, *p* < 0.001), ARCR < 3 cm (5.2 vs. 4.5 weeks *p* < 0.001) and >3 cm (5.9 vs. 5.1 weeks, *p* < 0.001), aTSA (4.9 vs. 4.3 weeks, *p* < 0.001), rTSR without subscapularis repair (4.0 vs. 3.6 weeks, *p* = 0.031), and isolated BT (3.7 vs. 3.3 weeks, *p* = 0.012) than Europe respondents. No significant differences between regions within the US and Europe were demonstrated. Conclusions: There is considerable variation in the immobilization advocated by surgeons, with geographic location and years of clinical experience influencing patterns of sling use. Future work is required to establish the most clinically beneficial protocols for immobilization following shoulder surgery. Level of Evidence: Level IV.

## 1. Introduction

Over 500,000 surgical procedures on the shoulder joint are performed each year in the United States (US) [1]. The number of shoulder replacements performed each year increased 5.6-fold between 1998 and 2017 [2], while surgery for rotator cuff tears has increased by approximately 10% each year over the last decade [3,4,5,6,7,8]. A significant determinant of recovery following shoulder surgery is the prescribed rehabilitation protocol [9,10,11,12,13,14,15,16,17].

A wide spectrum of postoperative rehabilitation protocols has been described for patients following open and arthroscopic procedures [18,19,20]. Surgeons may potentially improve outcomes after rotator cuff repair by controlling and optimizing the mechanical environment following surgery, with a period of immobilization being a widely adopted strategy [21,22,23,24,25]. Immobilization protects the shoulder from excessive forces that may damage the tissues or repair constructs and lead to early failure [26]. However, this must be balanced with the increased risk of postoperative shoulder stiffness and decreased shoulder function [26]. At present, the optimum duration of immobilization and its basic utility for a range of shoulder procedures remain unproven [27], with differing data and recommendations [21,22,23,24,25].

There is a wide range of shoulder immobilization products and protocols used by surgeons following shoulder surgery [25]. However, patterns of sling use among surgeons are not known and, furthermore, there is no consensus on the optimum position and duration of shoulder immobilization following a range of surgical procedures. The aim of this study was to establish patterns and types of sling use for various surgical procedures in the US and Europe and to identify factors associated with the variations. The hypothesis was that the types and durations of postoperative immobilization would vary depending on years of experience, procedures, and surgeons’ geographic locations.

## 2. Materials and Methods

**Figure 1:**
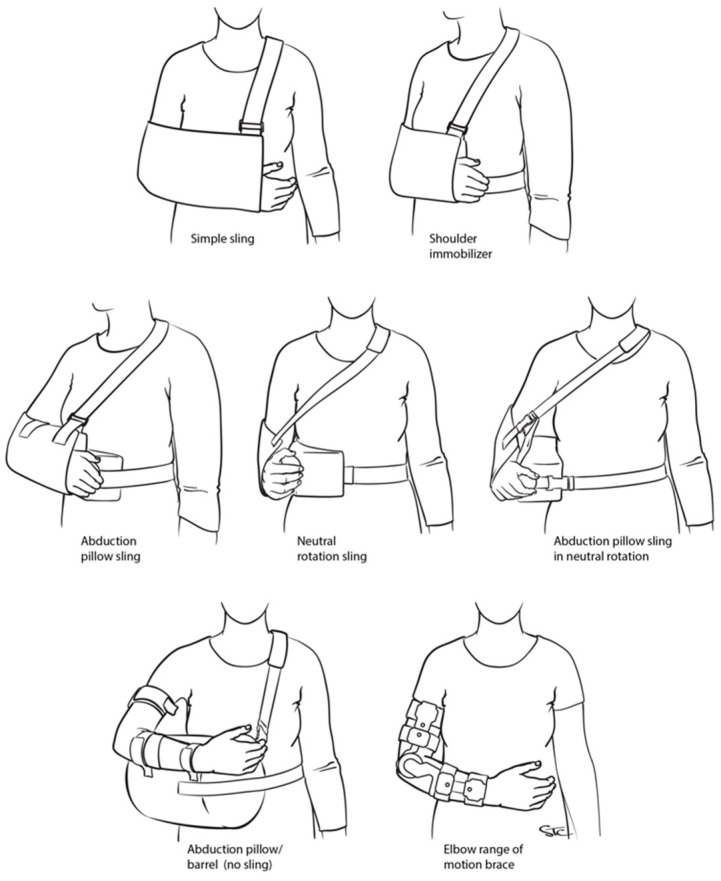
Selected methods of upper limb immobilization utilized following shoulder surgery.

An 18-question internet survey was developed and emailed to the membership of the American Shoulder and Elbow Society (ASES) and the European Society for Surgery of the Shoulder and Elbow (SECEC-ESSSE) in 20 April 2020 (Table 1). A reminder email was sent out on 6 May 2020. The data was downloaded and stored in a secure location at the Johns Hopkins University School of Medicine Department of Orthopaedic Surgery. Three of the 18 questions targeted demographic information, including country and region of practice and years of independent clinical practice. The type of immobilization was determined, including simple sling, shoulder immobilizer, abduction pillow sling, neutral rotation sling, abduction pillow sling in neutral rotation, and abduction pillow (no sling) (Figure 1). The remaining questions related to the technique and duration of immobilization following a variety of shoulder surgeries, namely arthroscopic Bankart repair, Latarjet procedure, arthroscopic superior/posterosuperior rotator cuff repair (ARCR) of tears <3 cm and >3 cm, anatomic total shoulder arthroplasty (aTSA) and reverse TSA (rTSA), and isolated biceps tenodesis.

Since this study did not rely on patient data but solely on doctors’ answers to an online survey, an a priori approval by an ethical committee or written informed consent was not required. However, the participants (doctors) agreed by answering the survey to use their answers for research purposes.

### Statistical Analysis

The responses were collected and tabulated using Microsoft Excel software version 16.69.1 (Microsoft Corporation, Redmond, WA, USA). Relationships between surgeon location and sling type were analyzed using Fisher’s exact tests and post-hoc tests using Bonferroni-adjusted *p*-values. Relationships between experience and sling duration were analyzed using Spearman’s correlation tests. The analysis was performed in RStudio (version 2022.12.0+353) (Posit Software, Boston, MA, USA) using a two-sided level of significance of 0.05.

## 3. Results

In total, 499 surveys were returned and completed. Some respondents did not answer some questions, and to maintain consistency, 499 was used as the denominator for all percentage calculations for the responses.

Of the 499 surgeon respondents, 54.7% were US-based, with 45.3% based in Europe (Table 2). The median years of experience in independent clinical practice was 15 (IQR = 9–25), with respondents from Europe being more experienced than respondents from the United States (median 18 vs. 12 years, *p* = 0.000095). The response rate was 273/992 = 28% (ASES) and 226/618 = 37% (ESSSE).

### 3.1. Arthroscopic Bankart Repair (ABR)

**Figure 2:**
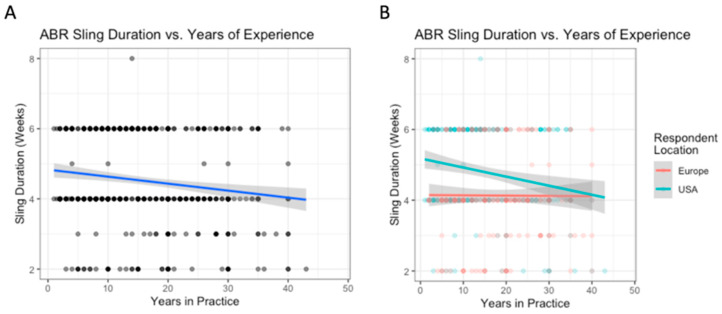
Arthroscopic Bankart repair: the relationship between years of experience and sling duration (**A**); with breakdown by location (**B**).

The variation in the use of sling based on surgeon location is outlined in Table 3. Surgeons based in the US reported higher abduction pillow sling use than Europeans (62% vs. 15%, *p* < 0.0001), while Europeans reported higher simple sling use than Americans (74% vs. 31%, *p* < 0.001) (Table 3). For each additional decade of experience, the average sling duration in weeks decreased by 0.2 weeks (Figure 2A). On average, respondents from the US reported one week longer sling durations than Europeans (mean 4.8 weeks vs. 4 weeks, *p* < 0.001). The interaction between location and experience was significant, indicating that the relationship between experience and sling duration was significantly different for US and European surgeons. Experience did not influence sling duration for European surgeons, but it did for US surgeons (Figure 2B). There were no significant differences between regions within the US and Europe.

### 3.2. Latarjet

**Figure 3:**
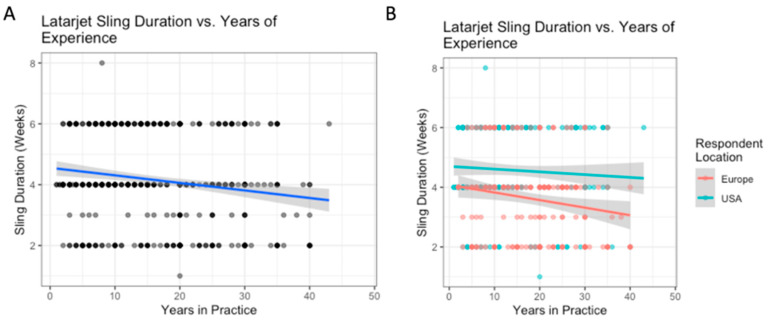
Latarjet procedure: the relationship between years of experience and sling duration (**A**); with breakdown by location (**B**).

The variation in use of sling based on surgeon location is outlined in Table 4. Surgeons based in the US reported higher abduction pillow sling use than Europeans (53% vs. 12%, *p* < 0.0001), while Europeans reported higher simple sling use than Americans (78% vs. 44%, *p* < 0.001) (Table 4). For each additional decade of experience, the average sling duration in weeks decreased by 0.25 weeks (Figure 3A). On average, respondents from the US reported 1 week longer sling durations than Europeans (mean 4.6 vs. 3.6 weeks, *p* < 0.001). The relationship between experience and sling duration was significantly different for US and European surgeons: experience influenced sling duration more for US surgeons than it did for European surgeons (Figure 3B). There were no significant differences between regions within the US and Europe.

### 3.3. ARCR (Tears < 3 cm)

**Figure 4:**
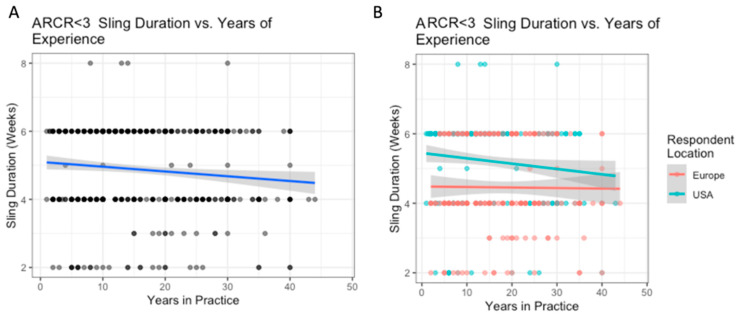
ARCR (tears < 3 cm): the relationship between years of experience and sling duration (**A**); with breakdown by location (**B**).

The variation in use of sling based on surgeon location is outlined in Table 5. Surgeons based in the US reported higher abduction pillow sling use than Europeans (80% vs. 42%, *p* < 0.0001), while Europeans reported higher simple sling use than Americans (50% vs. 17%, *p* < 0.001) (Table 5). For each additional decade of experience, the average sling duration in weeks decreased by 0.14 weeks (Figure 4A). On average, respondents from the US reported 1 week longer sling durations than Europeans (mean 5.2 weeks vs. 4.5 weeks, *p* < 0.001). There were no significant differences between regions within the US and Europe. The interaction between location and experience was significant, indicating that the relationship between experience and sling duration was significantly different for US and European surgeons. Experience did not influence sling duration for European surgeons, but it did for US surgeons (Figure 4B).

### 3.4. ARCR Cuff (Tears > 3 cm)

**Figure 5:**
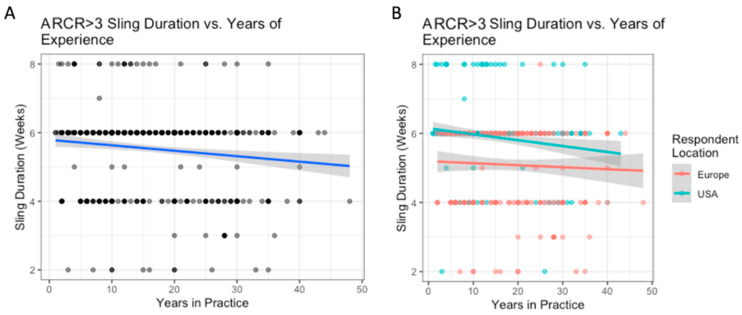
ARCR (tears > 3 cm): the relationship between years of experience and sling duration (**A**); with breakdown by location (**B**).

The variation in use of sling based on surgeon location is outlined in Table 6. Surgeons based in the US reported higher abduction pillow sling use than Europeans (84% vs. 61%, *p* < 0.0001), while Europeans reported higher simple sling use than Americans (34% vs. 13%, *p* < 0.001) (Table 6). For each additional decade of experience, the average sling duration in weeks decreased by 0.2 weeks (Figure 5A); however, experience did not influence sling duration for US and European surgeons when considered separately (Figure 5B). On average, respondents from the US reported 1 week longer sling durations than Europeans (mean 5.9 vs. 5.1 weeks, *p* < 0.001). Experience influences sling duration more for US-based surgeons than for Europeans (Figure 5B). There were no significant differences between regions within the US and Europe.

### 3.5. aTSA

**Figure 6:**
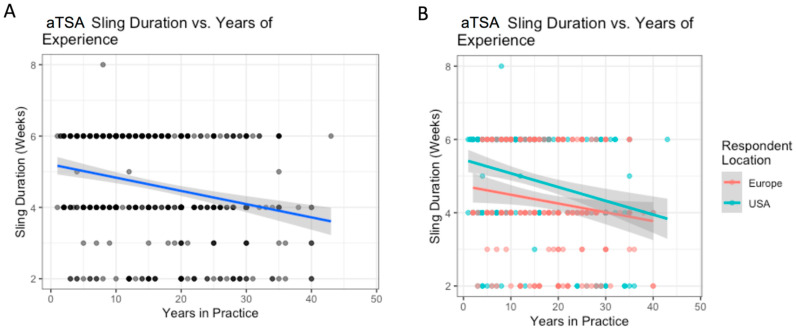
ATSA: the relationship between years of experience and sling duration (**A**); with breakdown by location (**B**).

The variation in use of sling based on surgeon location is outlined in Table 7. Surgeons based in the US reported higher abduction pillow sling use than Europeans (50% vs. 21%, *p* < 0.0001), while Europeans reported higher simple sling use than Americans (69% vs. 41%, *p* < 0.001) (Table 6). On average, respondents from the US reported 1 week longer sling durations than Europeans (mean 4.9 weeks vs. 4.3 weeks, *p* < 0.001). For each additional decade of experience, the average sling duration in weeks decreased by 0.4 weeks (Figure 6). However, the relationship between experience and sling duration was not significantly different between European and US-based surgeons. There were no significant differences between regions within the US and Europe.

### 3.6. rTSA with Subscapularis Repair

**Figure 7:**
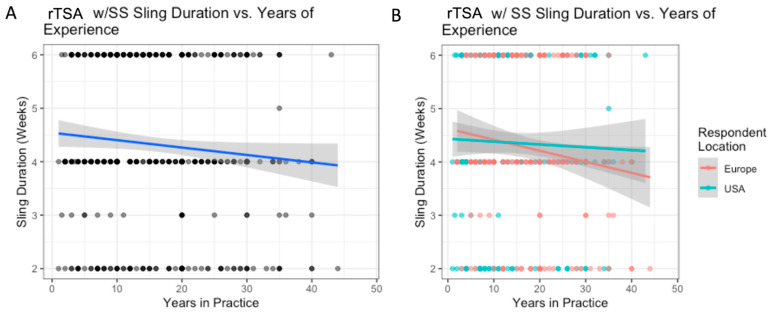
rTSA with subscapularis repair: the relationship between years of experience and sling duration (**A**); with breakdown by location (**B**).

The variation in use of sling based on surgeon location is outlined in Table 8. Surgeons based in the US reported higher abduction pillow sling use than Europeans (61% vs. 22%, *p* < 0.001), while Europeans reported higher simple sling use than Americans (69% vs. 35%, *p* < 0.001) (Table 8). There was no difference in sling durations between US and European respondents (mean 4.4 vs. 4.2 weeks, *p* = 0.414) and there were no significant differences between regions within the US and Europe. For each additional decade of experience, the average sling duration in weeks decreased by 0.1 weeks (*p* = 0.049) (Figure 7A). Experience did not influence sling duration differently for US and European surgeons (Figure 7B).

### 3.7. rTSA without Subscapularis Repair

**Figure 8:**
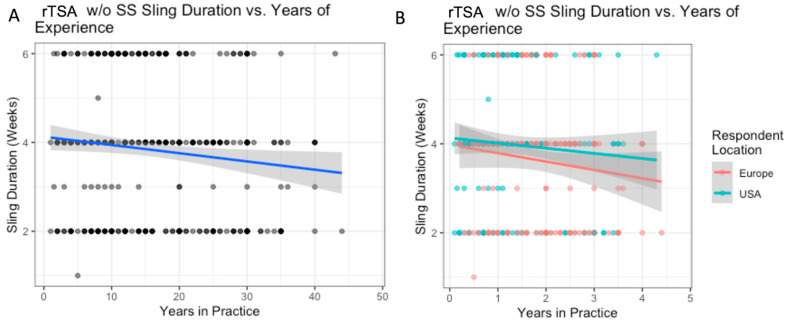
rTSA without subscapularis repair: the relationship between years of experience and sling duration (**A**); with breakdown by location (**B**).

Variation in use of sling based on surgeon location is outlined in Table 9. Surgeons based in the US reported higher abduction pillow sling use than Europeans (57% vs. 18%, *p* < 0.001), while Europeans reported higher simple sling use than Americans (73% vs. 39%, *p* < 0.001) (Table 9). For each additional decade of experience, the average sling duration in weeks decreased by 0.19 weeks (*p* = 0.022) (Figure 8A). Experience did not influence sling duration differently for US and European surgeons (Figure 7B). On average, respondents from the US reported 1 week longer sling durations than Europeans (mean 4.0 vs. 3.6 weeks, *p* = 0.031). There were no significant differences between regions within the US and Europe.

### 3.8. Isolated Biceps Tenodesis

**Figure 9:**
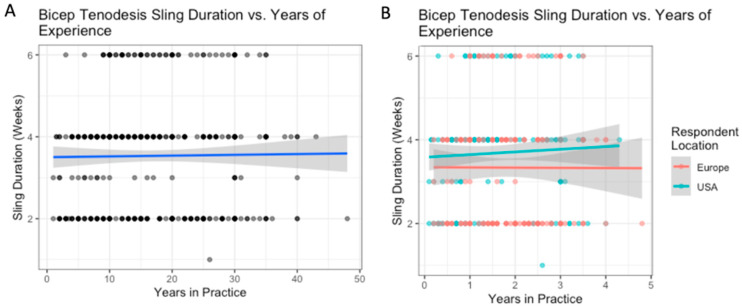
Isolated biceps tenodesis: the relationship between years of experience and sling duration (**A**); with breakdown by location (**B**).

The majority of respondents based in the US and Europe recommended simple sling immobilization following isolated biceps tenodesis procedures (77% and 82%, respectively, *p* = 0.899). However, respondents in the US reported higher abduction pillow sling use than Europeans (18% vs. 7%, *p* < 0.001) (Table 10). Experience did not influence the duration of immobilization within the US or Europe (Figure 9). There were no significant differences between regions within the US and Europe. US-based surgeons reported longer sling durations than their European counterparts. (3.7 vs. 3.3 weeks, *p* = 0.012). However, experience did not influence sling duration in either region.

## 4. Discussion

The most important finding of this study was the differences in preferred immobilization methods between US-based surgeons and their European counterparts. An abduction pillow sling was the preferred method of immobilization for the majority of US-based surgeons, following Bankart repair, Latarjet procedure, ARCR of tears <3 cm and >3 cm, aTSA, and rTSA. This is in contrast to European surgeons who preferred a simple sling for all procedures except for ARCR of >3 cm in size.

Availability and cost play an important role in postoperative immobilization. For instance, some abduction braces are either not available in Europe, simply not reimbursed by healthcare systems, or are sold at a prohibitive cost. Furthermore, regarding compliance with postoperative immobilization and abduction brace wearing, a publication revealed that about 50% of European patients actually do not adhere to the suggested immobilization regimen [28]. Moreover, some European publications advise the abolition of postoperative immobilization after Latarjet procedures, small to medium rotator cuff repairs, and rTSAs [23,29,30]. Consequently, surgeon selection of immobilization strategy may be influenced not only by the availability and cost of equipment and historical use of one method over another, but also by patient preference and compliance and recent publications, explaining differences found between the two continents.

There was also a considerable variation in sling durations recommended by respondents in both the US and Europe. Respondents recommended from 0–8 weeks of sling immobilization following Bankart repair, Latarjet procedure, ARCR of tears <3 cm and >3 cm, aTSA, and rTSA, and 0–6 weeks of immobilization following isolated biceps tenodesis. More experienced clinicians were more likely to recommend shorter periods of immobilization across all procedures surveyed, with the exception of isolated biceps tenodesis. For the majority of procedures, US-based surgeons reported longer sling durations than Europeans. This study highlights the considerable variation in practice among shoulder surgeons in postoperative immobilization. Currently, the optimum duration of immobilization and its basic utility for a range of shoulder procedures remains unproven [27] with differing data and recommendations [21,22,23,24,25]. Future work is required to establish the most clinically beneficial protocols for immobilization following shoulder surgery. This is challenging due to the range of immobilization strategies and durations currently being used.

This study has several strengths. This represented the largest study of shoulder surgeon specialists that the authors are aware of, with representation across all regions of the US and Europe. The surgeons who responded represent a comprehensive mix of experienced surgeons and surgeons just starting their practice. This study has several limitations, including the cross-sectional nature of the study, with responses based on surgeon opinion at a single point in time. As such, the study is subject to selection and recall bias. Additionally, although the number of responses was high, the response rate was low, which may introduce a potential source of bias pending the responses of those who did not participate, as well as those who chose to participate. Further, not all questions were answered by all respondents, likely due to survey fatigue, so a correction was needed in some cases to determine the proper percentages for the responses. In addition, using an online–only response system may have introduced selection bias by omitting potential responders who are less ‘technologically adept’.

## 5. Conclusions

There is considerable variation in the immobilization advocated by surgeons with geographic location and years of clinical experience influencing patterns of sling use. Future work is required to establish the most clinically beneficial protocols for immobilization following various types of shoulder surgery. This is challenging due to the range of immobilization strategies and durations currently being used.

## Figures and Tables

**Table 1:** The 18 question survey ^a^.

Survey Question
1. What country do you practice in?
2. If you practice in the United States, what region?
3. How many years have you been in practice?
4. Following arthroscopic Bankart repair- which shoulder immobilization technique ^a^ do you use?
5. For how long do they wear this?
6. Following arthroscopic superior rotator cuff repair < 3 cm- which shoulder immobilization technique ^a^ do you use?
7. For how long do they wear this?
8. Following arthroscopic superior rotator cuff repair > 3 cm- which shoulder immobilization technique ^a^ do you use?
9. For how long do they wear this?
10. Following a Latarjet procedure- which shoulder immobilization technique ^a^ do you use?
11. For how long do they wear this?
12. Following an anatomic total shoulder arthroplasty- which shoulder immobilization technique ^a^ do you use?
13. For how long do they wear this?
14. Following a reverse shoulder arthroplasty WITH subscapularis repair- which shoulder immobilization technique ^a^ do you use?
15. For how long do they wear this?
16. Following a reverse shoulder arthroplasty WITHOUT subscapularis repair- which shoulder immobilization technique ^a^ do you use?
17. For how long do they wear this?
18. Following an isolated arthroscopic or open biceps tenodesis (no cuff repair)- which shoulder immobilization technique ^a^ do you use?
19. For how long do they wear this?

^a^ No sling, simple sling, shoulder immobilizer, abduction pillow sling, neutral rotation sling, abduction pillow sling in neutral rotation, and abduction pillow (no sling).

**Table 2:** Location of surgeon respondent.

Practice Location	N (%)
United States	273 (54.7%)
Midwest	62 (23%)
Northeast	77 (28%)
South	73 (27%)
West	59 (22%)
Europe	226 (45.3%)
Eastern	12 (5%)
Northern	36 (16%)
Southern	60 (27%)
Western	118 (52%)

**Table 3:** Variation in use of sling following arthroscopic Bankart repair based on surgeon location.

	USA		Europe			
	N	%	N	%	*p*-Value	Post-Hoc Test
Abduction Pillow Sling	167	62%	34	15%	<0.001	<0.001
Neutral + Abduction	1	0%	2	1%		>0.999
Neutral Rotation Sling	4	1%	7	3%		>0.999
No Sling	2	1%	1	0%		>0.999
Shoulder Immobilizer	12	4%	11	5%		>0.999
Simple Sling	84	31%	163	74%		<0.001
Other	0	0%	2	0%		>0.999

Bonferroni adjusted *p*-value.

**Table 4:** Variation in use of sling following the Latarjet procedure based on surgeon location.

	USA		Europe			
	N	%	N	%	*p*-Value	Post-Hoc Test
Abduction Pillow Sling	137	53%	26	12%	<0.001	<0.001
Mayo	0	0%	1	0%		>0.999
Neutral + Abduction	0	0%	1	0%		>0.999
Neutral Rotation Sling	2	1%	6	3%		0.832
No Sling	1	0%	9	4%		0.044
Shoulder Immobilizer	5	2%	5	2%		>0.999
Simple Sling	115	44%	173	78%		<0.001
Sling if Pain	0	0%	1	0%		>0.999

**Table 5:** Variation in use of sling following the ARCR (tears < 3 cm) based on surgeon location.

	USA		Europe			
	N	%	N	%	*p*-Value	Post-Hoc Test
Ab Pillow No Sling	1	0%	1	0%	<0.001	>0.999
Abduction Pillow Sling	216	80%	108	42%		<0.001
Mayo	0	0%	1	0%		>0.999
Neutral Rotation Sling	2	1%	4	2%		>0.999
No Sling	1	0%	5	2%		0.805
Shoulder Immobilizer	5	2%	9	3%		>0.999
Simple Sling	45	17%	130	50%		<0.001

**Table 6:** Variation in use of sling following ARCR (tears > 3 cm) based on surgeon location.

	USA		Europe			
	N	%	N	%	*p*-Value	Post-Hoc Test
Abduction Pillow Sling	227	84%	136	61%	<0.001	<0.001
Neutral + Abduction	0	0%	1	0%		>0.999
Neutral Rotation Sling	3	1%	1	0%		>0.999
No Sling	2	1%	2	1%		>0.999
Shoulder Immobilized/Simple Sling	1	0%	0	0%		>0.999
Shoulder Immobilizer	3	1%	7	3%		>0.999
Simple Sling	35	13%	77	34%		<0.001

Bonferroni adjusted *p*-value.

**Table 7:** Variation in use of sling following aTSA based on surgeon location.

	USA		Europe			
	N	%	N	%	*p*-Value	Post-Hoc Test
Abduction Pillow Sling	132	50%	47	21%	<0.001	<0.001
External Rotation Brace	0	0%	1	0%		>0.999
Mayo	0	0%	1	0%		>0.999
Neutral Rotation Sling	3	1%	5	2%		>0.999
No Sling	11	4%	7	3%		>0.999
Shoulder Immobilizer	10	4%	8	4%		>0.999
Simple Sling	109	41%	154	69%		<0.001

**Table 8:** Variation in use of sling following rTSA with subscapularis repair based on surgeon location.

	USA		Europe			
	N	%	N	%	*p*-Value	Post-Hoc Test
Abduction Pillow Sling	155	61%	48	22%	<0.001	<0.001
External Rotation Brace	0	0%	1	0%		>0.999
Mayo	0	0%	1	0%		>0.999
Neutral Rotation Sling	2	1%	4	2%		>0.999
No Sling	3	1%	6	3%		>0.999
Shoulder Immobilizer	7	3%	5	2%		>0.999
Simple Sling	89	35%	153	70%		<0.001

**Table 9:** Variation in use of sling following rTSA without subscapularis repair based on surgeon location.

	USA		Europe			
	N	%	N	%	*p*-Value	Post-Hoc Test
Abduction Pillow Sling	148	57%	37	17%	<0.001	<0.001
Neutral Rotation Sling	2	1%	3	1%		>0.999
No Sling	2	1%	20	9%		<0.001
Shoulder Immobilizer	6	2%	0	0%		0.150
Simple Sling	100	39%	161	73%		<0.001

**Table 10:** Variation in use of sling following isolated biceps tenodesis based on surgeon location.

	USA		Europe			
Sling	N	%	N	%	*p*-Value	Post-Hoc Test
Abduction Pillow Sling	49	18%	16	7%	<0.001	0.002
Elbow ROM Brace	1	0%	0	0%		>0.999
Neutral Rotation Sling	1	0%	2	1%		>0.999
No Sling	10	4%	19	9%		0.192
Shoulder Immobilizer	3	1%	3	1%		>0.999
Simple Sling	209	77%	183	82%		0.899

## Data Availability

The following was the link sent out to all members of the American Shoulder and Elbow Society and the European Society for Surgery of the Shoulder and Elbow in April of 2020. https://docs.google.com/spreadsheets/d/1iCKrhYmPvbhTiYyXcAdd5-Obiw1E6NUPVMXymYQKEAI/edit?usp=sharing (accessed on 21 January 2023). The initial survey was sent out on 20 April 2020 and a reminder email was sent out on 6 May 2020. The data was downloaded and stored in a secure location at the Johns Hopkins University School of Medicine Department of Orthopaedic Surgery.
